# Subsidence of the Cholera in Europe and Asia

**Published:** 1848-05

**Authors:** 


*Subsidence of the Cholera in Europe and Asia.*—Intelligence has been received from St. Petersburgh up to the date of the 29th January (17th, O. S.) The following remarks on the subject of the cholera have been published by Doctor Thielmann in the Russian Medical Gazette.

During the month of December the severe cold so completely arrested the progress of Asiatic cholera, that there was reason to believe it would disappear entirely. It has altogether ceased in the provinces around the Caspian ; and with the exception of Moscow, Mohilew, and Witepsk, it is no longer met with in any of the great cities or towns of the empire. Even in these, and in smaller places, the disease has assumed so mild a character, that it appears to be on the point of extinction.

Letters from Constantinople of the 1st of January announce the gradual disappearance of cholera in that city. The epidemic was then chiefly confined to the Arsenal ; and out of 210 attacked, only 58 died. Accounts from Bagdad of the 7th of December state that the cholera had almost entirely disappeared from Kerkoula and Suleymania. Letters from Mossol, dated the 12th of December, mention that the cholera had ceased in that city, after having killed 300 persons; and intelligence from Aleppo of the 18th, states that it has appeared at Beregik, on the banks of the Euphrates, and was causing from ten to fifteenth deaths daily.

On the whole this intelligence is highly favourable.

*Dublin Medical Press.*

